# Food access and associated socioeconomic factors in the Durham-Chapel Hill Metropolitan Statistical Area (MSA), North Carolina

**DOI:** 10.1371/journal.pone.0330333

**Published:** 2025-09-05

**Authors:** Zahra Al Hamdani, Matthew Jansen, Tia Marie Francis, Philip McDaniel, Lisa Jo Melnyk

**Affiliations:** 1 Department of Environmental Sciences and Engineering, 135 Dauer Drive, University of North Carolina- Chapel Hill, North Carolina, United States of America; 2 University Libraries, Davis Library, 208 Raleigh St, University of North Carolina- Chapel Hill, North Carolina, United States of America; Public Library of Science, UNITED KINGDOM OF GREAT BRITAIN AND NORTHERN IRELAND

## Abstract

The adversity of diet-related diseases is increased because of food insecurity . North Carolina is higher than the national average (11.7%) in food insecurity at 13.9%. The availability of healthy foods in households depends on the spatial access within the food environment where people reside or work. This study characterized the food environment, food access and associated socioeconomic factors in the Durham-Chapel Hill metropolitan statistical area (MSA) at a census block group level. Using GIS and statistical techniques, the average weighted median (AWM) was devised as measure of access for food outlets; associations between the AWM and socioeconomic variables were then investigated using multivariate regressions. For everyone in the MSA, the analysis showed lowest accessibility for fruit and vegetable markets (AWM = 2), and the highest accessibility for restaurants (AWM = 136). Relative to the White population, percentage point increase in the African American population in a block group led to a statistically significant increase in access to all categories of food outlets, with the highest increase in access of fruits and vegetable markets at 4% (p < 0.001). For every person increase in household size a decrease there was a decrease in the AWM of fruit and vegetable markets and food banks by 40%. The approach used in this study can be used in across localities measure access at a higher geographic granularity (block group level) and the associated sociodemographic factors. The results highlight disparities in food access which may require public health interventions.

## Introduction

The Food and Agriculture Organization of the United Nations defines Food security as “the situation that exists when all people, at all times, have physical and economic access to sufficient safe and nutritious food that meets their dietary needs and food preferences for an active and healthy life” [1]. Pothukuchi (2015) presented another definition that accounts for additional elements- they defined food security as the “access at all times to high quality, affordable, and culturally acceptable food through means that maximize social justice and environmental sustainability” [2]. Food security is an issue that caught the attention of the policy makers in the United States. According to statistics from the United States Department of Agriculture (USDA), it is estimated that over 40 million Americans suffer from low accessibility to healthy food [3]. Since access to healthy food is inherent to food security, it can be further deduced that 40 million Americans are food insecure.

The adversity of diet-related diseases is increased because of food insecurity [4]. This is because individuals who are food insecure tend to depend on foods that are high- energy and low cost [5]. Such dietary patterns can result in chronic, diet-related diseases like diabetes, obesity and hypertension, and even mental health conditions [6,7].

Food access, a component of food insecurity, is multidimensional- researchers refer to it as the physical access to stores or other purchasing locations, the affordability of foods, and the in-store availability [8]. In turn, the availability of healthy foods in households, like fruits, vegetables, low- fat dairy products, and grains, is largely dependent on the spatial access to the food available in the neighborhood, i.e., the neighborhood food environment [9].

Neighborhood socioeconomic characteristics are an essential piece in food access research. Studies have shown that lower-income neighborhoods have a higher likelihood of having access to convenience stores rather than supermarkets, as compared to their higher-income neighborhood counterparts [10–13]. Furthermore, studies have shown racial and ethnic differences in food access, whereby communities of color in comparison to majority- White communities have higher access to fast- food restaurants and convenience stores and less access to supermarkets [12,14–17]. The differential access to foods amongst communities, based on differing neighborhood characteristics, can give rise to health and disease disparities. Further exploring these socioeconomic, neighborhood characteristics can help identify the specific, disparity- causing factors and in turn lead to targeted public health interventions [18].

Studies of food environments define neighborhoods as census tracts or ZIP codes, which, due to their large size, serve as a poor proxy [19]. To better understand the food environment at a neighborhood level, it is important to characterize the area and examine potential disparities in availability and access at a finer, higher- resolution geographic scale or in simpler terms, at a scale that gives more of a neighborhood “feel”. Thereby in this study, a finer geographic space namely the census block group was used as a proxy for a neighborhood.

North Carolina (NC) is one of the top 10 agricultural states in the United States [20], with sales of agricultural products in 2022 valued $18.7 billion giving it the 8th rank nationally [21]. Yet, North Carolina is higher than the national average (11.7%) in food insecurity at 13.9% [22], with 1 in 5 children facing hunger regularly [23]. Highlighting food access, an element of food insecurity, is important in understanding a contributing factor to diseases like obesity and diabetes in NC. Prior research of food access and its association with neighborhood census characteristics, such as, racial/ethnic composition is limited at a finer geographic granularity in North Carolina beyond census tracts, such as block groups. The purpose of this study was to (1) systematically characterize the food environment and food access of a localized area in North Carolina at a block group level, namely the Durham-Chapel Hill Metropolitan Statistical Area (MSA), and (2) to determine if food access is correlated to neighborhood characteristics such as race, income, and education level. In the course of answering these objectives, an approach was developed for this NC neighborhood that can be adapted to other defined neighborhoods in order to identify potential food deserts.

## Methods

### Ethics statement

The Office of Human Research Ethics at the University of North Carolina – Chapel Hill, upon review of the proposed work and as per the definition under the United States federal regulations, deemed that the research does not require Institutional Review Board (IRB) approval since no human subjects were recruited.

### The study area

Within the study area, a census block group was deemed a proxy for ‘neighborhood’. The smallest census areas for which income data is available are block groups [24], which is a key component for measuring neighborhood socioeconomic status. As per the U.S Census Bureau (2022), the population size of a block groups is in the range of 600 and 3,000 people.

**Fig 1:**
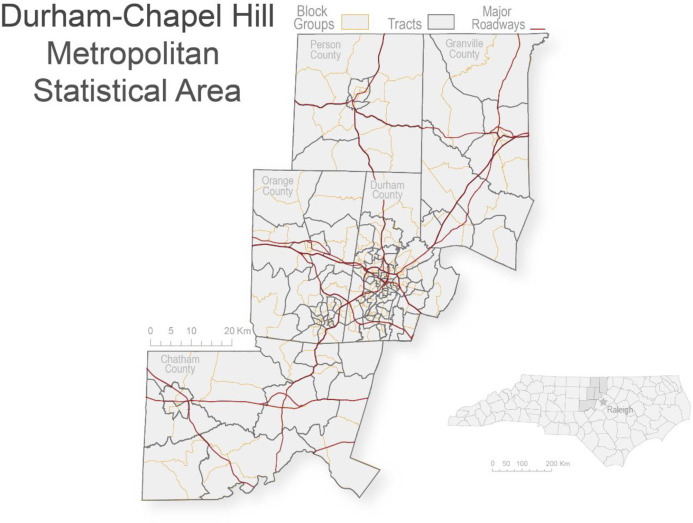
Study area: Durham–Chapel Hill Metropolitan Statistical Area (MSA) [25–28].

The study area consisted of 321 block groups across counties in North Carolina that compose the Durham- Chapel Hill MSA (Fig 1). The five counties are Chatham, Durham, Granville, Orange, and Person, with a total population of 645,559 people as of 2019, across a land area of 5,931 square kilometers [29].

Metropolitan statistical area (MSA) is the term used to refer to a geographical area comprised of a city and surrounding communities connected via social and economic factors [30]. MSAs can consist of 1 or more counties. The centroid of the MSA is the county or counties that include extensively urbanized areas [31]. In this study, those counties are Durham and Orange. The counties surrounding the centroid and with which it has at least 25% commute interchange are also included in the MSA, with this interchange considered an indicator of “metropolitan integration” [31].

**Fig 2:**
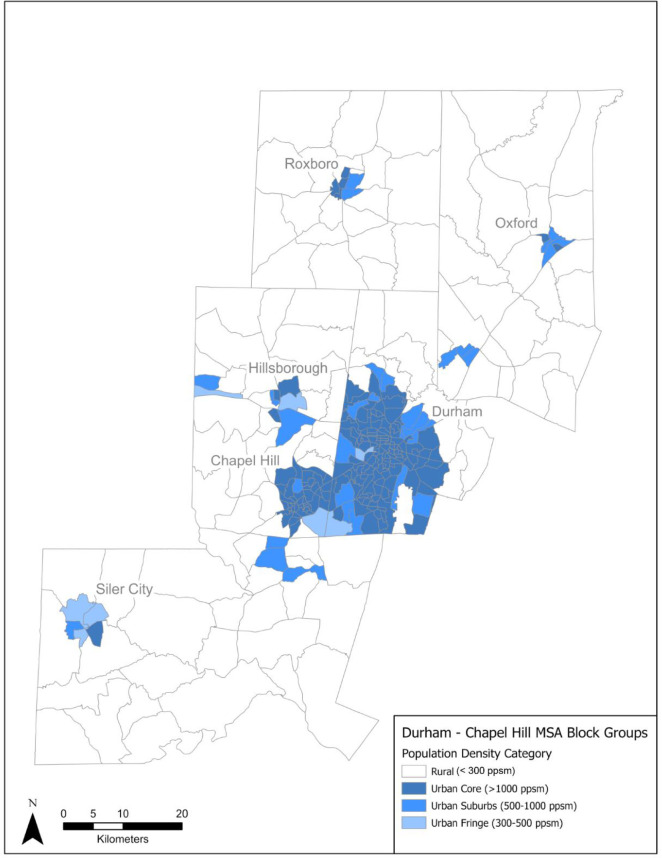
Population density in the Durham–Chapel Hill MSA.

Fig 2 shows the urbanicity breakdown of the Durham-Chapel Hill MSA using population density as defined by the U.S. Census Bureau. The Bureau uses 1,000 persons per square mile (ppsm) as the threshold for urban cores, and 500 ppsm to fill out the territory [32]. As an extension to these definitions, two more categories of population density were added: urban fringe (300–500 ppsm) and rural (<300 ppsm).

Studying an MSA, as opposed to a single county, permits consideration of metropolitan integration in relation to food access. For example, residents may live at a county border and shop in the neighboring county or may live in a county and shop on the way back from work. The latter is a typical population dynamic in Durham and Chapel Hill, the main cities in Durham and Orange counties respectively. Residents of Durham may work in Chapel Hill and shop for food in Chapel Hill on their commute back home.

### Demographic factors

The American Community Survey five-year data for the years 2014–2018 [29], retrieved from the distributor ‘social explorer’, was the source of the demographic variables selected a priori for this study. The following were the socioeconomic and sociodemographic factors (and associated table numbers from the survey) assumed to be correlated to the food environment in the Durham- Chapel Hill MSA and included in the regression analysis: population density was used as a proxy of urbanicity (A00002), racial breakdown (A03001), education level (A12002), and household income (A14006), age of the neighborhood structures indicated by the year the structures were built (A10057), and the owner occupancy (A10045).

### Food environment

The food outlets in the Durham- Chapel Hill MSA where food can be obtained through purchase comprise the food environment in this study. The only exception was food banks or food pantries where food need not be purchased to be obtained. Data on food outlets was retrieved from the Reference Solutions database [33]. The Reference Solutions database is created and maintained by a private company, Data Axle, which conducts telephone surveys to gather information on individual businesses. Information includes company name, complete address, latitude and longitude coordinates for mapping, the Standard Industrial Classification (SIC), and the North American Industry Classification System (NAICS) 6-digit numbered codes that are used to define the type of business, franchise and brand information, among other details [34].

Table 1 shows the food outlet types, the corresponding NAICS codes, the definition of each of these food outlets, and examples of each of the food outlet types in North Carolina.

**Table 1:** Food outlet classification, Durham-Chapel Hill MSA.

Food store category	NAICS codes	NAICS codes description	Examples from North Carolina
Grocery stores and Superstores	445110, 452311	Outlets that retail general groceries like frozen and canned foods, fruits, vegetables, dairy products, meats, poultry, and other perishable groceries	Harris Teeter, Whole Foods, BJs
Specialty food stores	4452− 10, 20, 30, 99	Outlets that retail specialized lines of food like ethnic foods, meat, fish and seafood markets.	Carolina Fish Market, Southeast Oriental Market
Convenience stores, Confectionary, and nut stores	445120, 447110, 445292	Outlets that retail a limited range of food items such as bread, milk, soda and other snacks. Confectionary and nut stores are outlets engaged in retailing candy, chocolate, lollipops and other confections, nuts, and popcorn	7- Eleven, Sheetz, Godiva Chocolatier, Candy factory
Food banks	624210	This food outlet type includes the establishments that collect, prepare and deliver food for those in need. Examples include: food banks, soup kitchens and meal delivery programs.	Food Bank- Central-Eastern NC
Fruit and Vegetable markets	445230	Outlets that retail fresh fruits and vegetables.	Mountain Fresh Produce, Farm Fresh Markets
Restaurants	722410, 7225− 11, 13, 14, 15	Outlets that include limited-service restaurants (Also referred to as fast food restaurants), full- service restaurants and drinking places, cafes, delis	Cheesecake Factory, McDonald’s

### GIS methodology

In ArcGIS Pro (ArcGIS Pro 2.8, Redlands, CA), a GIS layer of block groups for the study area was used, along with the Federal Information Processing Standard (FIPS) code used to uniquely identify each block group. A 3-mile buffer was created around the MSA to capture accessible food outlets both within the study area and its periphery [35]. This considered the food outlets that are adjacent to the MSA and accessible to the communities within the MSA, which would otherwise be lost if the geographic border of the MSA were used as the boundary. The Reference Solutions business locations, including the latitude and longitude coordinates of the food outlets, were overlaid on the census block group layer. The road network was, used to create service areas around food outlets, which are territories that represent areas that are a 10-minute travel time by car from every food outlet. Using the road network and its components, such as direction, and speed limits, in our calculation provides a plausible simulation of how people reach food destinations as they drive to work or home, for instance. An assessment of the spread of food outlets in the study area in showed an abundance of food outlets within a 10-minute drive, and therefore, the assumption was that 10 minutes was a reasonable travel time for a food destination to be considered accessible. It was expected that a longer drive time would lead to greater accessibility and therefore a more conservative, yet reasonable travel time of 10 minutes was assumed. Furthermore, previous research discussed that low-income families travel an average of 1.58 miles to food outlets in urban neighborhoods [36], which corresponds to a travel time of approximately 10 minutes. Another study mentioned that the average distance traveled between homes and food stores in five U.S. cities is 2.6 miles [37]. These cities were Los Angeles, California; Chapel Hill, North Carolina; Albuquerque, New Mexico; Columbus, Ohio; and Philadelphia, Pennsylvania. It is assumed that these distances would be traveled in 10 minutes or less.

**Fig 3:**
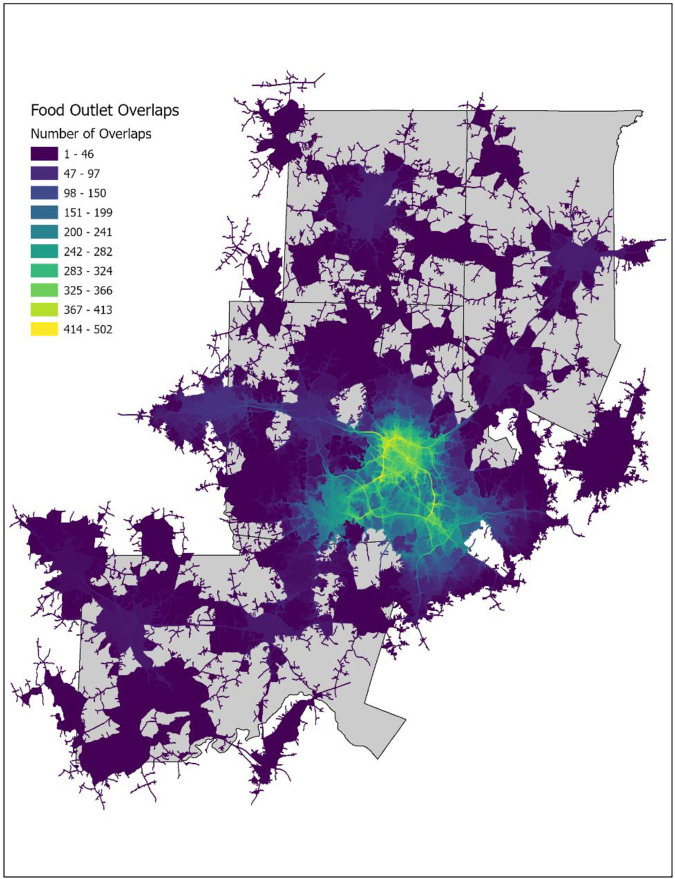
Food outlet overlaps in the Durham–Chapel Hill MSA.

After creating the service areas, the overlapping buffers were counted to determine how many food outlets serve any given area within the MSA (Fig 3). This allows a quantitative measure of both the presence and quantity of food outlets across the study site. For each block group in the MSA, the percentage that was covered by zero 10-minute food outlet buffers, one 10-minute food buffer, two 10-minute food buffers, up to the maximum number of overlaps within the block group, was calculated. Following the tabulations, this allowed the determination of the relative abundance or scarcity of food resources within each block group. Table 2 (and the stylized schematic) show this calculation for a sample block group in the study area.

**Table 2:** Calculating the Area Weighted Median (AWM) for Grocery Stores in Block Group with FIPS 370370201031.

Count of grocery stores	Area, in square miles	Percentage of total block group area	Cumulative Percentage of total block group area
0	8.0	17	17
1	2.2	8.7	26
2	0.5	1.9	28
3	5.1	20	48
**4**	**4.9**	**19**	**67**
5	1.8	7.2	74
–			
–			
–			
19	0.3	1.1	100

### Statistical analysis

Table 2 includes the data that the GIS analyses generated for grocery stores in one block group in Chatham County as an illustrative example of the food access calculations. The steps described here were carried out for all categories of food outlets for all the block groups across the MSA. FIPS is the unique spatial identifier for every block group, ‘count of grocery stores’ is the number of overlapping 10-minute buffers within each block group, and ‘area’ represents the land area that is covered by the overlapping buffer territories. The grocery stores could be in neighboring block groups but still accessible. Dividing ‘area’ by the total area of the block group generates the ‘percentage’ of the block group area covered by the respective ‘count’ of grocery stores. As the name implies, the ‘cumulative percentage’ was computed by cumulatively adding the values in the ‘percentage of total block group area’. The area weighted median (AWM) is the count such that 50% of the area can get to at least that many stores and the other 50% can get to at most that many stores. In this example, 4 grocery stores are within a 10-minute drive of 67% (cumulative percentage) of the block group’s area, and, therefore, 4 is the AWM for this FIPS code. In summary, 4 is the first value over 50% cumulative.

The area weighted median was used instead of a simple count as a measure of food access to account for variability across the geographic area. For example, 5% of the area of one block group may be within 10 minutes of 20 grocery stores, but 50% of that block group is within 10 minutes of 5 grocery stores only, 20% only has 2 grocery stores, and so on. A mere count of the food outlets across the study area has the underlying assumption that the food stores are uniformly distributed and equally accessible to the population. The weighted median was assumed to be a realistic measure of food store access since it simulates real travel by incorporating the road network in the area. The 10-minute travel time incorporates the location of each food store, its occupied area, and its intersection with the block groups.

Spearmen correlations were used to evaluate the bivariate relationship between each of the demographic variables of the block groups and access to the food outlets, represented by the AWMs for each food store category. The Benjamini-Hochberg procedure [38,39] was used as a false discovery rate correction. This procedure decreased the chances of a “false positive,” in this case, a false significant correlation between any two variables, and is usually deployed when multiple correlations are completed at the same time. The p values for the Spearman correlations are adjusted by running the Benjamini-Hochberg procedure and are more reliable. The Spearman correlations were a probing step to examine whether any significant associations existed between the variables under study and was followed by a multivariate regression. These correlation and regression analyses were run using R (R 4.1.0, Vienna, Austria). The demographic factors were the independent variables in the model and the outcome variables were the categories of food outlets (Table 1), namely: grocery stores and superstores, specialty food stores, convenience stores, food banks, fruit and vegetable markets, and restaurants. Instead of using population density as is, the natural log of the values was taken; this step greatly improved the fit of the models as captured by the lower values of the Akaike Information Criterion (AIC) of the models. The best model was chosen based on an overall goodness of fit from a finite set of count regression models, namely: Poisson regression, zero inflated Poisson regression, zero inflated Poisson regression, negative binomial regression and zero inflated negative binomial regression. Due to the large number of zeros in the raw data, zero inflated models were included in the model candidate list.

To compare the performance of the two non-zero inflated models, specifically the Poisson and negative binomial regressions, the likelihood ratio test was used. This is a model selection tool intended to compare two non-nested models. The null hypothesis was that both models perform equally well; the null hypothesis is rejected at low p values. The same test was applied to compare the two zero inflated models to one another. The Vuong test with Bayesian Information Criterion (BIC) adjustment [40] was used to compare the performance of the zero-inflated models to the non-zero-inflated models. The null hypothesis for this test was that the two models were indistinguishable, to be rejected at low p values.

## Results

### Description of the demographics of the study area and its food environment

The following summary statistics are provided at the block group level (n = 321) and are based on the American Community Survey (ACS) 2014–2018 ACS 5-Year Estimates. The average population density per square mile was 2130 (standard deviation (SD): 2,664). The average of the median household income per block group was $63,323 (SD: $29,38). The average population with a bachelor’s degree or more at 43% (SD: 25). The average household size per block group was 2 (SD:0.40) and the average home age was 37 years (SD:15 years). Average percent White (non-Hispanic) was 56% (SD:26%); African American (non-Hispanic) was 26% (SD:22%); Hispanic was 11%(SD:12%), and other races was 7% (SD:8%) per block group. Other races included those accounted for in the American Community Survey 2018 and are: Asian, Native Hawaiian and other Pacific Islander, Native American or Alaska Native, and two or more races (U.S. Census Bureau, 2019). Owner-occupied housing was at 61% (SD:27%).

**Fig 4:**
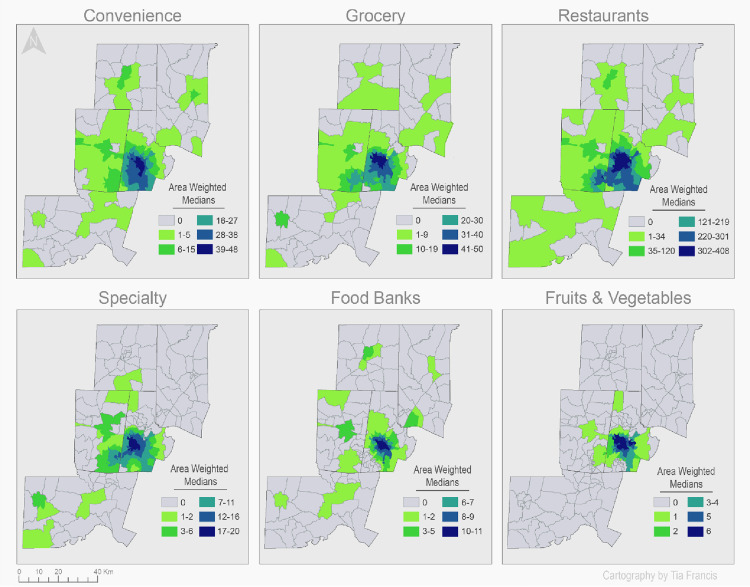
Area Weighted Medians (AWM) of food outlet accessibility across block groups.

Table 3 and Fig 4 present the characteristics of the food environment in the Durham-Chapel Hill MSA. The total number of food outlets across the study area was 2,017. Table 3 shows the striking difference between the accessibility of restaurants and that of fruit and vegetable markets, both in total counts and in the average of the weighted medians: 152 average number of restaurants and 2 fruit and vegetable markets per block group, and 1,519 and 13 in total counts, respectively. Notably, the restaurants are predominantly fast-food, as determined by the prevalent NAICS code (722511) for these businesses, under the restaurants category. Grocery stores and supermarkets are the second most accessible food stores, albeit at a wide range: 18 grocery stores and 152 restaurants per block group. Specialty stores and food banks have relatively low food store accessibility at average weighted medians of 6 and 3, respectively.

**Table 3:** Food environment in Durham- Chapel Hill MSA (n = 321 block groups).

Food store category	Total count in MSA + 3-mile buffer	Average of the area weighted medians (standard deviation)/ block group
Restaurants	1,519	152 (136)
Grocery stores and supermarkets	206	18 (16)
Fruit and vegetable markets	13	2 (2)
Food banks and pantries	31	3 (3)
Convenience stores	178	16 (15)
Specialty food stores	70	6 (6)

In line with the food environment description in Table 3, Fig 4 provides a visual of the distribution and access of food outlets across the Durham- Chapel Hill MSA where lighter greens indicate a lower number of outlets. Fruit and vegetable markets are the least accessible and are mainly located in and around the Durham urban center as represented by the higher AWMs and dark blue shaded area, with accessibility decreasing as the distance increases from the Durham city center. Some block groups in the MSA, namely in Person, Chatham, and Granville counties, are completely devoid of fruit and vegetable markets. The same pattern is seen with food banks and specialty stores. However, they are more accessible compared to the fruit and vegetable markets as reflected by the higher AWMs shown by the increased shaded areas. Restaurants, predominantly fast-food restaurants, are the most accessible across the Durham-Chapel Hill MSA and not only around the Durham urban center. Still, the highest AWMs for restaurants are in Durham County. A similar pattern is seen for convenience and grocery stores, in that higher AWMs for these store categories relative to fruit and vegetable markets and cover more of the MSA as demonstrated by the increased shaded areas.

The Spearman calculations summarized in Table 4 show positive correlations between the access of all categories of food outlets and population density, home age, the African American and Hispanic populations at a block group level. Out of the original 321 block groups of the study area, full demographic information was available for 303 block groups and therefore the number of block groups included in the multivariate regression is 303 rather than 321. In relation to all demographic variables, population density has the strongest positive correlation with access of all food outlet categories. This is both reasonable and expected; more food outlets are needed to cater to larger populations per square mile. The strength of the correlations varied across the food outlets; it was strongest between population density and access to grocery stores and supermarkets, restaurants were a tight second, and weakest with the AWM of food banks at 0.85, 0.84 and 0.65, respectively. Home age had a strong positive correlation with the access of all food outlets although relatively weaker than population density. The strongest correlation of home age was with the AWM of food banks at 0.43, and the weakest with specialty food stores at 0.32 (p < 0.001).

**Table 4:** Correlation matrix of demographic variables and food outlets in the Durham- Chapel Hill MSA (n = 303, block groups).

	Restaurants	Grocery stores and supermarkets	Fruits and Vegetable Markets	Food banks	Convenience stores	Specialty food stores
**Population density, per square mile**	0.84*	0.85*	0.66*	0.65*	0.82*	0.78*
**Household income, in 1000s**	−0.18	−0.23*	−0.24*	−0.36*	−0.24*	−0.18
**Education level: % bachelors degree or more**	0.38*	0.31*	0.17	0.01	0.23*	0.34*
**Race/ ethnicity: % White (non Hispanic)**	−0.38*	−0.42*	−0.38*	−0.52*	−0.51*	−0.35*
**Race/ ethnicity: % Hispanic**	0.31*	0.36*	0.30*	0.33*	0.36*	0.30*
**Race/ ethnicity: % African American (non-Hispanic)**	0.19*	0.22*	0.25*	0.42*	0.36*	0.15
**% Other races/ ethnicities**	0.34*	0.29*	0.17	0.05	0.22*	0.32*
**Household size**	0.29*	−0.22*	−0.19	−0.15	−0.23*	−0.24*
**Home age**	0.35*	0.37*	0.33*	0.43*	0.33*	0.32*
**Owner occupied**	−0.61*	−0.62*	−0.49*	−0.51*	−0.59*	−0.57*

*p value <0.001.

Some races were also positively correlated to the access of food outlets. The African American population had the strongest correlation with the AWM of food banks at 0.42 (p < 0.001) and the weakest correlation with the AWM of specialty stores at 0.15, albeit statistically non-significant. The Hispanic population had its strongest correlations with the AWM of both grocery stores and supermarkets as well as convenience stores at 0.36 (p < 0.001), and the weakest with the AWM of both fruits and vegetable markets and specialty stores, both at 0.30 (p < 0.001).

On the other end of the spectrum, negative correlations were observed between all categories of food outlets and the percentage of White (non-Hispanic) population, household income, household size, and owner occupancy of housing units at a block group level. Owner occupancy of housing units had the strongest negative correlation with the access of food outlets (p < 0.001). The strongest negative correlations were with the AWM of grocery stores and supermarkets at −0.62 (p < 0.001) and restaurants at −0.61 (p < 0.001). The weakest was for the AWM of fruits and vegetable markets at −0.49 (p < 0.001). The weakest correlation with owner occupancy is still stronger than all the correlations of food access with household income and household size. The AWM of food banks was negatively correlated with household income at −0.36 (p < 0.001), and the weakest correlation with household income was with the AWM of restaurants tied with specialty stores at −0.18, however statistically non-significant.

Multivariate regressions generated coefficients whose exponential values are the incidence rate ratios (IRR) shown in Table 5. The IRRs are a measure of the magnitude and direction of the association of the demographic variables with the various food outlet types at a block group level in the MSA. Values greater than 1 mean that a positive relationship holds between the variables in question and values less than 1 mean that the relationship is negative. For example, the coefficient of the regression between household income and fruits and vegetable outlets is −0.001. Taking the exponent of this coefficient, exp(−0.001), generates the IRR between household income and fruits and vegetable outlets, 0.999. The IRRs are centered around 1 and not zero because the IRRs are values that are exponentiated, thereby never 0.

**Table 5:** Associations of the demographic variables with the number and category of food store outlets in the Durham- Chapel. Hill MSA (n = 303 block groups).

	*Negative binomial models IRR (CI)*		*Zero-inflated negative binomial models IRR (CI)*			
Demographic variables	Food Banks	Fruit & Vegetables	Restaurants	Convenience	Grocery & supermarkets	Specialty
	(1)	(2)	(3)	(4)	(5)	(6)
Log of population density, per square mile	1.57* (1.34, 1.80)	1.77* (1.41, 2.04)	2.00* (1.79, 2.22)	1.62* (1.46, 1.79)	1.50* (1.38, 1.63)	1.25* (1.38, 1.63)
Median household income, in 1000s	1.00 (0.992, 1.01)	1.00 (0.991, 1.01)	1.01 (1.00, 1.01)	1.01 (1.00, 1.01)	1.00 (0.100, 1.01)	1.00 (0.100, 1.01)
% With Bachelors or more	1.01 (0.998, 1.02)	1.02* (1.01, 1.03)	1.02* (1.01, 1.03)	1.01 (1.00, 1.02)	1.02* (1.01, 1.02)	1.012* (1.01, 1.02)
% Hispanic	1.03* (1.02, 1.04)	1.04* (1.03, 1.06)	1.03* (1.02, 1.04)	1.02* (1.02, 1.03)	1.02* (1.02, 1.03)	1.02* (1.01, 1.02)
% African American (non-Hispanic)	1.02^*^ (1.01, 1.03)	1.02^*^ (1.02, 1.03)	1.02^*^ (1.01, 1.02)	1.02^*^ (1.01, 1.024)	1.01^*^ (1.01, 1.02)	1.01^*^ (1.01, 1.02)
% Other races	1.01 (0.992, 1.03)	1.02^*^ (1.00, 1.04)	1.01 (0.998, 1.03)	1.01 (0.995, 1.02)	1.01 (1.00, 1.02)	1.01 (1.00, 1.02)
Household size, in persons	0.60^*^ (0.402, 0.806)	0.60 (0.377, 0.826)	0.69 (0.525, 0.852)	0.62^*^ (0.484, 0.754)	0.80 (0.659, 0.944)	0.86 (0.694, 1.02)
Home age, in years	1.03^*^ (1.02, 1.03)	1.03^*^ (1.02, 1.04)	1.01 (1.00, 1.02)	1.01^*^ (1.01, 1.02)	1.01^*^ (1.01, 1.02)	1.02^*^ (1.01, 1.02)
% Owner Occupancy	1.01 (0.999, 1.01)	1.01 (0.998, 1.02)	1.00 (0.996, 1.01)	1.00 (0.996, 1.01)	1.00 (0.995, 1.00)	1.00 (0.993, 1.00)

*Note:* ^*^p < 0.001.

Similar to the inferences from the Spearman correlations, population density had IRRs that were both large in magnitude and positive in direction. Population density had the highest IRR with restaurants compared to all other food outlets. This implies that for the same increase in population density in a block group there is a bigger increase in the access of restaurants compared to fruit and vegetable markets, grocery, convenience, specialty stores and food banks. Every log increase in the population density leads to a 100% increase in restaurant AWMs, or a 100% increase in restaurant access, 77% for fruit and vegetable markets; 62% for convenience stores, food banks and grocery stores are expected to increase by a comparative 57% and 50%, respectively, and the lowest increase in access is for specialty stores by 25%, all at p < 0.001.

The values shown in Table 5 indicate that race had a statistically significant impact on food outlet access in all categories at a block group level. With the White population as the referent population, every percentage point increase in the Hispanic population in the Durham- Chapel Hill MSA, was associated with an increase in the AWM of fruit and vegetable markets by 4% (p < 0.001). In other every percentage point decrease in the White population led to a 4% increase in access to fruit and vegetable markets. The second highest increase in access was that of food banks and restaurants at 3% (p < 0.001). Specialty, convenience, and grocery store access all increased in the same magnitude of 2% for every percentage point increase in the Hispanic population (p < 0.001) at a block group level. Similar results were observed for the African American population. A percentage point increase in the African American population, or a decrease in the White population relative to the African American population in a block group, resulted in a statistically significant increase in access to all categories of food outlets. The AWM of food banks, restaurants, convenience stores and fruit and vegetable markets increased by 2%, followed by grocery and specialty stores at 1% for every percentage point increase in the African American population at a block group level.

Home age and household size impacted food access, albeit in opposite directions. With every one person increase in average household size in the block group, a statistically significant decrease was noted in food access across food categories. This decrease was most prominent on food banks and fruit and vegetable markets: for every person increase in household size a decrease was observed in the AWM of both fruit and vegetable markets and food banks by 40%. A decrease was observed in the AWMs of convenience stores and restaurants by 38% and 31% (p < 0.001), respectively.

On the other hand, increase in home age led to an increase in access for all food categories in a block group, with the highest increase being for fruit and vegetable markets and food banks at 3% in the AWM for every year increase in home age at a block group level. The AWM increases for restaurants, grocery and convenience stores was the same; for every year increase in the home age an increase of 1% was observed in these three categories. This value was topped by the increase in the AWM of specialty stores at 2%, all at p < 0.001.

## Discussion

The U.S. Department of Agriculture’s Food Access Research Atlas and other research studies like Morland et al. (2002b), Block et al. (2004) and Zenk et al. (2005b) have explored food access in the U.S. at a tract level [17,41,42]. Limited research has assessed food access and its associated socioeconomic variables at a granular neighborhood level in North Carolina localities. This cross-sectional study highlighted the components of the food environment, and measured food access and the correlated socioeconomic variables in the Durham-Chapel Hill MSA. It is important to point out that the associations highlighted between the socioeconomic variables in this study and food access do not indicate causation but rather an association. The results demonstrated that the MSA population in general could be suffering from some level of food insecurity relative to the low access of grocery stores and even lower access of fruits and vegetable outlets whose presence is considered an indicator of availability of healthy food. Despite the grocery stores being the most accessible after restaurants, there is a large difference in AWM for these two categories: 152 AWM for restaurants that are primarily fast food and 18 AWM for grocery stores per block group. There were 2 AWM for fruit and vegetable outlets per block group. On average, 4 out of the 5 counties that make up the MSA were completely devoid of fruit and vegetable outlets per block group. In this case, food insecurity may not be the absence of sources of healthy food altogether but instead the abundance of non-nutritious food, as per the definition of the Food and Agriculture Organization of the United Nations [1].

Our findings also extend prior work in the region. Major et al. (2018) analyzed food access in Durham using a relative healthy food access (RHFA) metric based on the proportion of healthy to unhealthy outlets at the census tract level [43]. While that approach helped identify general patterns, it relied on a binary outlet classification and coarser spatial units. In contrast, our study examines a broader geographic area (Durham-Chapel Hill MSA), distinguishes among six food outlet types, and uses a 10-minute drive-time network analysis at the block group level. These enhancements allow for a more detailed and spatially precise understanding of access disparities, particularly in areas with mixed land use and variable road connectivity.

Based on the findings of this study, much of the concentration of accessible food in the MSA is in Durham County which puts the populations living farther from Durham Country block groups at a food access disparity. According to Blanchard and Matthews (2007) supermarkets and grocery stores being mainly concentrated within some towns and cities rather than being widespread geographically is a high concern that contributes to food inaccessibility across the U.S [44]. They noted that affordable and high quality foods are often concentrated in what they referred to as “retail centers” such as Durham County, and subsequently subjecting individuals and communities living outside these “retail centers” to low access to such foods [44].

Evidence of strong associations between socioeconomic variables at a census block group level and food access was shown to be prevalent in the study area. Among the socioeconomic factors that are related to disparity in food access is education. Across 862 census tracts in Montreal, Canada, fruit and vegetable stores were disproportionately more accessible to participants with higher education [18]. This aligned with the results of this study where with every percentage increase in the population of people with a bachelor’s degree or more, an increased access of fruit and vegetable stores was observed.

Racial disparities in food access are not uncommon in the U.S. A study conducted in 216 tracts across various states namely: Maryland, Minnesota, Mississippi and North Carolina showed that White majority neighborhoods had 4 times the frequency of supermarkets compared to their African American majority neighborhoods [45]. Similarly, Raja et al. (2008) showed that supermarkets were completely absent in neighborhoods with predominantly colored populations compared to neighborhoods with predominantly White populations [19]. An interesting finding from Morland et al. (2002a) was that White majority neighborhoods had a lower prevalence of grocery stores and convenience stores [45]. This study found a similar pattern where access to convenience stores, restaurants and grocery stores increased for every percentage point increase in the African American population relative to the White population.

A contrast was seen with other studies exploring racial differences in food availability. These studies found that neighborhoods with predominantly African American and Latino populations had a lower prevalence of fresh produce in comparison to corner and convenience stores; the opposite pattern was observed in neighborhoods with predominantly White populations [12,13,46–48]. This study showed that population increase in the African American or Hispanic population led to increased access for all categories of food outlets, including fruit and vegetable markets. Interestingly, the highest access increase was that of fruit and vegetable markets for both African American and Hispanic population increases. Similarly, Moore et al. (2006) also showed that predominantly African American neighborhoods in Forsyth County, North Carolina had significantly more fruit and vegetable markets and grocery stores than White neighborhoods [49]. Going up north to Erie County, New York a network of small grocery stores were available in neighborhoods with predominantly colored populations [19]. In a national study that looked at 28,050 zip codes across the U.S., Powell et al. (2007) showed that minority and low-income neighborhoods had a higher prevalence of non-chain supermarkets and small grocery stores [13]. Differences in the local geography and food environment results in differences in the populations who are at an access disparity. In this setting, Durham County and especially the city of Durham is the major center for food stores of all types as evident by the maps in Fig 4, and is also the major center of employment opportunities, and hence is likely to attract newcomers. By comparison, counties like Chatham, Person and Granville offer relatively fewer economic opportunities, be it industrial or agricultural. The established population of these latter counties have lived there for generations and are probably more likely to commute longer distances for work than to relocate to Durham, whereas immigrants who are likely Hispanic populations would move directly to where the jobs are, i.e., Durham. This may explain the unexpected positive correlation between the Hispanic population and access of all food store types, and especially with access to fruit and vegetable outlets and grocery stores.

A study conducted across156 tracts in New Orleans, Louisiana, it was found that predominantly African American neighborhoods were at a disparity of higher access of unhealthy foods due to the higher density per square mile of fast food restaurants relative to neighborhoods with majority White populations [41]. Kwate et al. (2009) results also found that in five boroughs in New York City which were predominantly African American, there was a higher fast food density relative to predominantly White majority areas [50]. This study found that an increase in the African American and Hispanic populations resulted in a statistically significant increase in access to restaurants, which are predominantly fast food- in line with the disparity in unhealthy food access in New Orleans, Louisiana.

In this study, maps were created as visual snapshot to aid in the analysis of food access in the Durham-Chapel Hill MSA. The methodology of development of these maps could adopted by researchers and policymakers as a screening tool to identify areas that could be potential food deserts and could be easier to disseminate information on access to the public. Such tools could complement other tools like the EPA’s EnviroAtlas or the Food Environment Index, with the benefit that the maps in this study provided data at a finer geographic granularity due to analysis conducted at a block group level. For a deeper analysis beyond screening, the study presented a detailed characterization of the different categories of food outlets that comprise the food environment, and their access at a census block group level. Implications from the study would assist food security advocates, local governments, community representatives, policy makers and other stakeholders in drawing a more detailed picture of what the food environment and food access looks like in their local geographies. Regressions with socioeconomic variables provided in-depth information regarding food access correlations in the Durham- Chapel Hill MSA and, therefore, shed light on some associations of food access disparity, which could result in chronic health illnesses disparities.

This research is not void of limitations that should be accounted for to produce finer-tuned results in future food access work. The factors included in the regression are not exhaustive: vehicle ownership, public transportation use, and walkability are neighborhood characteristics that may offer additional insights. This study evaluates food access as a function of spatial proximity of food outlets to residential block group. Given that food access is multidimensional, not encompassing other dimensions of food access such as in-store variability and affordability is a limitation. In addition, results may be prone to a systematic error due to miscounting of food outlets. If one or more of the food store categories are over-represented or under-represented in the Reference Solutions database this would lead to reduced precision. [13]. For example, some healthy food vendors like fresh produce stands informally available to the locals may not be accounted for in commercial databases like Reference Solutions which would in turn lead to the undercounting of outlets under the fruit and vegetable category. Lastly, a 10-minute travel time assumption is a short period in rural settings; residents of rural areas are likely accustomed to longer travel times which would thereby impact their access. Still, the results of this study share similarities with numerous previous research which speaks to the strength of the methodology despite the limitations. It is also important to remember that assumptions like travel times and buffer zone distances can be changed in this approach, thereby making it adaptable to any study area in question, and allows for its use beyond the Durham-Chapel Hill MSA

Future studies of food access could include in store environment of the various food store outlets discussed in this study in an effort to capture the multidimensionality of food access. Some components that could be measured for the in-store food environments are availability and variety [51]. Availability is based on high consumption foods, while variety refers to the availability of at least two alternatives of foods and beverages [51]. This method has been adopted for the Chilean Ministry of Health in their efforts to assess the store food environments in the country [51]. Another method for in store food environment assessment would be to use the instrument developed by Glanz et al. (2007) namely the Nutrition Environment Measure Survey for Stores [52]. This tool is used to measure quality, availability and price of food items across 13 categories like baked goods, beverages, fruits and vegetables, milk, frozen dinners etc. making NEMS a widely used retail food assessment tool [53].

In order to close the gap between the actual number of food outlets and that is in commercial databases, an on-the-ground- team of researchers can be deployed to count the number of outlets per food outlet category in the study area, a method used in other research like Gordon et al. (2011) [54]. Every food retail outlet and vendor, both formally or informally available to local residents, would be screened in the Durham-Chapel Hill MSA. The surveyors would then determine the type of establishment and the location of each outlet, prior to giving a final count of each food store type across the study area. The shortfall of this method however is the substantial amount of time that would be needed to cover the study area, in addition to having a larger team of surveyors which would entail higher research cost.

## Conclusions

Policymakers are investigating the correlation between the disparities in the occurrence of chronic, diet-related diseases like diabetes and obesity and neighborhood food access and availability [54]. A systematic approach was developed to target the food environment in a North Carolina locality, to measure food access and correlation to SES neighborhood factors. Given that food security and its correlation with health issues is a national concern, this approach can be transferred and used in other localities to investigate food environments and access at fine geographic granularity as is much needed. In examining the food store access and associated factors at the census block group level, the study found that disparities in access exist in the Durham-Chapel Hill MSA. A clearer and easy-to-disseminate understanding of how neighborhood-level factors influence food access is essential to (1) supplement environmental justice efforts that target reducing disparity in food access and availability, (2) provide tools that would spread awareness within neighborhoods on the state of their food access, disparity and the impacting factors, and (3) inform health interventions and guide evidence-based policy that aims at reducing chronic diseases at a neighborhood level, which would ultimately improve the health state of families and communities at large.

## Supporting information

S1 File Supplementary Tables. Contains block group–level data for food outlet categories in the Durham–Chapel Hill MSA. Includes: **S1 Table** (convenience stores), **S2 Table** (food banks), **S3 Table** (fruit and vegetable outlets), **S4 Table** (grocery stores), **S5 Table** (restaurants), **S6 Table** (specialty stores), and **S7 Table** (example Area Weighted Median calculation for grocery stores).
(ZIP)

S2 File Median Abundance Dataset. Block group–level dataset of Area Weighted Median (AWM) accessibility values for food outlet categories (e.g., restaurants, grocery stores, convenience stores) in the Durham–Chapel Hill MSA.
(CSV)
